# Fatal Measles without Rash in Immunocompetent Adult, France

**DOI:** 10.3201/eid1803.111300

**Published:** 2012-03

**Authors:** Julien Lupo, Sylvain Bernard, Claire Wintenberger, Monique Baccard, Astrid Vabret, Denise Antona, Jean-François Timsit, Patrice Morand

**Affiliations:** Centre Hospitalier Universitaire de Grenoble, Grenoble, France (J. Lupo, S. Bernard, C. Wintenberger, M. Baccard, J.-F. Timsit, P. Morand);; Université Joseph Fourier, Grenoble (J. Lupo, J-.F. Timsit, P. Morand);; Centre Hospitalier Universitaire de Caen, Caen, France (A. Vabret);; Institut de Veille Sanitaire, Saint-Maurice, France (D. Antona)

**Keywords:** measles, acute respiratory distress syndrome, rash, death, France, viruses

**To the Editor:** The reemergence of measles in Europe is a reminder of the forgotten risk for severe illness and death associated with this disease in industrialized countries. Since 2008, >20,000 measles cases and 9 measles-associated deaths (in 7 immunocompromised and 2 immunocompetent persons) have been reported to the French Institute for Public Health. Among these cases, the reported causes of death were pneumonia and/or acute respiratory distress syndrome (ARDS) (n = 7) and encephalitis (n = 2). All patients except 1, an immunocompromised patient, had the typical morbillous rash. We report another fatal case of measles, with intractable ARDS but no rash, in an apparently immunocompetent adult.

The patient was a 29-year-old woman in Grenoble, France, who smoked but had no relevant medical history except an episode of depression. In 2011, she sought care for fever, cough, coryza, diarrhea, and a 10-kg weight loss over 10 days. A general practitioner empirically prescribed pristinamycin and oral prednisone (60 mg/d for 5 d) for sinusitis. Five days later, the patient was admitted to the hospital because of persistent signs and symptoms. Physical examination at admission (day 1) detected fever (38.5°C), dyspnea, and a low body mass index of 17.5 kg/m^2^. Hematologic tests showed nonregenerative anemia (hemoglobin concentration 9 g/dL) and leukopenia (2.2 × 10^9^ leukocytes/L) with profound lymphopenia (0.2 × 10^9^ lymphocytes/L) and mild thrombocytopenia (135.0 × 10^9^ platelets/L). A chest radiograph showed bilateral diffuse interstitial infiltrates. Antimicrobial therapy with levofloxacin and ceftriaxone was started.

On day 2, several examinations were conducted to explore the possibility of underlying immunosuppressive disease. Body scans showed no adenopathy or lesions suggestive of cancer. HIV test result was negative. General immunologic test results were within normal limits (immunoglobulin quantification, autoantibody testing) or consistent only with an acute viral infection (serum protein electrophoresis). A bone marrow biopsy sample indicated isolated erythroblastopenia with no abnormality of other cell lineages (PCR for parvovirus B19 was negative).

On day 3, because of severe respiratory failure, the patient was transferred to the intensive care unit, where the diagnosis of ARDS was confirmed and mechanical ventilation was started. Treatment with tazocillin/tazobactam, ciprofloxacin, amphotericin B, and acyclovir was also started. Microbiological findings from bronchoalveolar lavage (BAL) samples were repeatedly negative for bacteria, mycobacteria, fungi, and *Pneumocystis jirovecii*. Cytology of BAL samples showed an acute inflammatory response with atypical epithelial cells, supporting a diagnosis of viral infection. However, none of 14 respiratory viruses or human herpesviruses type 1, 3, 4, 5, or 6 were recovered from BAL samples by PCR. Blood and urine culture results were repeatedly negative, as were serologic test results for *Legionella* spp., *Mycoplasma pneumoniae*, and *Chlamydia pneumoniae*.

On day 5, because of refractory ARDS, venoarterial extracorporal membrane oxygenation was started. On day 6, the results of a broad serologic investigation demonstrated isolated IgM against measles virus. The patient was additionally given ribavirin, corticosteroids, and intravenous immunoglobulin. On day 10, the lymphocyte level had returned to reference range and the anemia had become regenerative. However, the patient’s respiratory condition did not improve, and after 2 weeks of the oxygenation therapy, the patient died of hemorrhagic shock. Her parents declined an autopsy.

PCR testing of the patient’s saliva by the French National Reference Center confirmed the presence of measles virus. Retrospective testing of serum, bone marrow, and BAL specimens collected during days 2–20 of hospitalization demonstrated measles virus RNA. The strain was identified as genotype D4, which is the epidemic strain circulating in France and elsewhere in Europe (*1*). The patient had no history of enanthem (Koplik spots) or morbilliform rash before or after symptom onset and no documented history of measles vaccination.

Deaths from measles with pneumonia or ARDS but without rash have been reported but mostly in patients with deficient cell-mediated immunity (*2**–**6*). Despite all our testing, we found no indications of an underlying immunosuppressive disease in this patient; however, we cannot categorically rule out this possibility, especially that of a primary immunodeficiency. The initial therapy with corticosteroids and the patient’s weight loss could also have interfered with her cellular immune response. The diagnosis of ARDS caused by measles was supported by detection of the measles genome in BAL samples and body fluids in the absence of any other pathogen, but pulmonary superinfection with unidentified pathogens could not be ruled out. Detection of the measles genome and isolated erythroblastopenia in the bone marrow biopsy sample is consistent with reports that measles virus can infect erythroid progenitors and interfere indirectly with hematopoiesis (*7**,**8*). Although ribavirin and passive immunotherapy have been reported to aid in recovery from severe measles pneumonia, their clinical efficacy is still unproven (*9**,**10*); and for the patient reported here, they were probably used too late.

This unusual case underscores the need for physicians to consider the diagnosis of measles, even in the absence of classical clinical features, during measles outbreaks. It also reemphasizes the insufficient vaccination coverage against measles in France.
